# 

**Published:** 1895-06

**Authors:** 


### CONTENTS.

ORIGINAL ARTICLES:

Tuberculosis. By AUSTIN PETERS, V.S. . . . . . . . . . . . 337

Horseshoeing. By J. H. FERSTER, V.S. . . . . . . . . . . . 348

SELECTIONS:

Subcutaneous Injections of Turpentine in Pneumonia—Affections of the Heart and Acute Founder—Tuberculosis—Glanders—The "Horse Industry" in Kansas—Influenza Organism—Antitoxin . . . . . . . . . . . 355

EDUCATIONAL:

Honors to Veterinary Educators—The Minneapolis Board of Trade Milk and Meat Inspection Meeting . . . . . . . . . . . . . . . . 369

EDITORIALS:

Our New Associate Editor and Owner—Good Roads—New York Veterinary Medical Association—So-called "American Sheep Disease"—Growth of Veterinary Literature—Pennsylvania Points the Way—Association of Faculties of Veterinary Colleges of North America—United States Veterinary Medical Association 371

NEW PUBLICATIONS: . . . . . . . . . . . . . . 376

SOCIETY PROCEEDINGS:

Keystone Veterinary Medical Association—Veterinary Medical Association of New York County—Reunion of Graduates of the American Veterinary College—New Hampshire Veterinary Medical Association—Gossip . . . . . 379

LEGISLATION:

France—Pennsylvania—Massachusetts—New York—Maryland—Aspect of New Legislation . . . . . . . . . . . . . . . . 382

CLINICAL GLEANINGS—CORRESPONDENCE—NOTES OF CASES—NECROLOGY—AUTOPSIES—FACTS AND ACTS WORTH KNOWING—NEW INVENTIONS—U. S. V. M. A.—COLLEGE NEWS—PERSONAL . . . . . . 386–402

